# How to Diagnose and Treat CD5-Positive Lymphomas Involving the Spleen

**DOI:** 10.3390/curroncol28060390

**Published:** 2021-11-11

**Authors:** José Cabeçadas, Victor E. Nava, Joao L. Ascensao, Maria Gomes da Silva

**Affiliations:** 1 Department of Pathology, Portuguese Institute of Oncology Lisbon, 1099-023 Lisboa, Portugal; jcabecadas@ipolisboa.min-saude.pt; 2 Department of Pathology, The George Washington University, Washington, DC 20037, USA; victor.nava@va.gov; 3 Department of Pathology, Veterans Health Administration Medical Center, Washington, DC 20422, USA; 4 School of Medicine, The George Washington University, Washington, DC 20037, USA; ascensao4@msn.com; 5 Department of Hematology, Portuguese Institute of Oncology Lisbon, 1099-023 Lisboa, Portugal

**Keywords:** low-grade, B-cell lymphoma, splenic lymphoma, CD5, immunophenotype, differential diagnosis

## Abstract

Patients with CD5-expressing lymphomas presenting with splenomegaly are frequently diagnosed with chronic lymphocytic leukemia. The most important differential diagnosis is mantle cell lymphoma, both in its classical and leukemic, non-nodal forms, given its prognostic and therapeutic implications. Other small B-cell neoplasms that frequently involve the spleen and occasionally express CD5 include the splenic marginal zone lymphoma, hairy cell leukemia and, rarely, lymphoplasmacytic lymphoma. The frequency of CD5 positivity depends in part on the sensitivity of the detection methods employed. Usually, a combination of morphological, immunophenotypic and molecular findings allows for a precise sub-classification of CD5-positive, low-grade B-cell lymphomas of the spleen. Some of these tumors may display a mixture of small and larger B cells, raising the possibility of more aggressive lymphomas, such as diffuse large B-cell lymphomas (DLBCL). Approximately 5–10% of DLBCL are CD5-positive and some may manifest as primary splenic lesions. When available, the morphology of DLBCL in the splenic tissue is distinctive and a leukemic picture is very rare. In conclusion, the appropriate morphological and clinical context assisted by flow cytometry panels and/or immunohistochemistry allows the differential diagnosis of CD5-positive, non-Hodgkin, B-cell lymphomas involving the spleen.

## 1. Introduction

As a secondary lymphoid organ, the spleen is frequently involved in lymphoid tumors [1]. Splenomegaly, while usually accompanied by other disease manifestations, may be the presenting feature. In these cases, particular issues are raised during the diagnostic workup, with an impact on further clinical decisions and management. Careful laboratory evaluations, including CBC, PB smear examination, phenotypic analysis and bone marrow (BM) biopsy with immunohistochemistry (IHC) may be instrumental for the correct identification of the underlying disease [2,3,4]. Nowadays, diagnostic splenectomy is reserved for cases where other methods fail to identify a specific entity [5,6]. Splenic biopsies, although feasible, carry the risk of hemorrhage and may not always be representative [7,8].

The expression of CD5 on B-cell neoplasms is classically indicative of chronic lymphocytic leukemia (CLL), an indolent neoplasm, or mantle cell lymphoma (MCL), a usually aggressive lymphoma [9]. Commonly, the expression of Cyclin D1 is present only in MCL, allowing the precise differentiation and appropriate treatment of these entities. However, overlapping conditions exist, which demand more sophisticated diagnostic techniques. Although rare, CD5 positivity can also be present in other, mostly indolent, lymphomas, including splenic marginal zone lymphoma, hairy cell leukemia and lymphoplasmacytic lymphoma [1,2,9,10,11]. Aggressive lymphomas are rarely localized exclusively in the spleen. Among these, diffuse large-cell, B-cell lymphoma (DLBCL) is the most frequent subtype and shows a positivity for CD5 in less than 10% of cases [12]. Other aggressive lymphomas that may occasionally express CD5 and affect the spleen include the intravascular large B-cell lymphoma and the T-cell/histiocyte-rich B-cell lymphoma, a subtype of DLBCL. The final diagnosis of these extra-nodal, aggressive B-cell lymphomas is typically made during the examination of splenectomy specimens since the involvement of PB or bone marrow (BM) tends to be sparse.

Splenic involvement detected by imaging is more sensitive than clinical examination. Splenomegaly is defined as ≥13 cm in splenic craniocaudal length assessed by CT [13]. However, an enlarged spleen is not equivalent to lymphomatous involvement, and lymphoma can be present in normal-sized spleens. FDG-PET/CT has become the preferred method for the staging and detection of splenic involvement in FDG-avid non-Hodgkin lymphomas, including DLBCL and follicular lymphoma, despite their radiotracer heterogeneous uptake [13]. CD5-positive indolent lymphomas, however, are not included in this group, except when transformed. PET-CT findings may be difficult to interpret when nodular lesions are absent. A diffuse pattern of infiltration, also seen in reactive conditions, requires skillful interpretation, mostly if the organ is not enlarged [14]. 

Of note, even in the absence of an overt leukemic picture, indolent CD5-positive B-cell lymphomas frequently involve the BM and/or PB to a degree that allows diagnostic sampling. Therefore, the careful examination of a PB smear and flow cytometric analysis should be undertaken as the first diagnostic step in the heterogeneous group of splenic lymphomas.

## 2. Clinical Vignette–Part 1

A 69-year-old man is referred to the outpatient hematology clinic with abdominal discomfort and a recently discovered mild leukocytosis, without other symptoms. A clinical examination reveals a palpable spleen 5 cm below the rib cage, confirmed by an abdominal ultrasound. No superficial lymph nodes are palpable. Complete blood counts (CBC) show 14 g/dL hemoglobin with 86 fL MCV, white blood cells 12,000/µL with 45% lymphocytes and 185,000/µL platelets. Other laboratory tests including lactate dehydrogenase (LDH), beta-2-microglobulin levels, kidney and liver panels are normal. The peripheral blood (PB) smear is inconspicuous except for the presence of occasional, mildly enlarged lymphocytes with condensed chromatin and irregular nuclei.

A lymphoid neoplasm is suspected. As a first diagnostic step, flow cytometry testing is ordered to characterize the PB lymphocytes.

## 3. Chronic Lymphocytic Leukemia (CLL)

### 3.1. Definition

CLL, the most common lymphoid neoplasm in the Western world, has a yearly incidence of 5/10^5^ people and is predominant in Caucasian males, with an increasing incidence with age [2]. The disease is characterized by the accumulation of small, mature CD5- and CD23-positive mature B cells, usually in the blood, BM and secondary lymphoid organs, including lymph nodes and spleen. According to the International Workshop on CLL (iwCLL) criteria, a minimum of 5000 monoclonal B lymphocytes/µL are required for diagnosis [15]. Cases with lower counts correspond to the premalignant Monoclonal B-cell Lymphocytosis category, provided no cytopenias, enlarged lymph nodes and/or hepatosplenomegaly or other extra-nodal involvement are present. A small lymphocytic lymphoma (SLL) is morphologically, immunophenotypically and biologically indistinguishable from CLL and should be diagnosed in cases sparing the PB, based on morphology and immunophenotype; this method is more commonly conducted for examining lymph node biopsy [2,15]. Although rare, CLL may manifest as an isolated splenomegaly that triggers the investigation of an underlying lymphoid tumor [1,16,17].

### 3.2. Clinical Features

CLL/SLL usually presents with leukocytosis and lymphocytosis, with or without lymphadenopathy and hepatosplenomegaly. Cytopenias occur in advanced stages or, less often, as a consequence of hematological autoimmune manifestations in 5–10% of patients [15,18,19]. Progressive hypogammaglobulinemia is the rule [20]. Other characteristics include a significant immune dysregulation manifesting as an increased susceptibility to infections and relatively frequent occurrence of second tumors. Patients presenting solely with splenomegaly are rare [16,17], and more frequently lymphadenopathy is identified during the diagnostic workup. Current recommendations for CLL management do not require imaging exams [15]. However, the presence of splenomegaly, isolated or accompanied by a lymphocyte count close to the upper normal limit, should prompt the radiological staging and biopsy of an accessible lymph node.

### 3.3. Microscopy, Immunophenotype and Genetic Profile

In a PB smear, CLL cells typically appear as small mature lymphocytes with a scant cytoplasm and a clumpy chromatin, without evident nucleoli. Some larger, atypical cells and prolymphocytes may also be seen, usually representing less than 10% of lymphocytes. If the percentage of such cells surpasses 55% of total the lymphocytes then the diagnosis of prolymphocytoid transformation should be considered [2]. Flow cytometry characterization is mandatory, and frequently sufficient for a definitive diagnosis [2,9,11,18]. The canonical immunophenotype includes the expression of pan-B-cell markers (CD19, CD20, CD22, CD79b) together with CD5, CD23 and LEF1, and a negativity for FMC7 and CD10. However, CD23 may be lacking or be expressed below the level of detection. The weak expressions of CD20, CD22, CD5 and surface light chains is the norm. In doubtful cases, the positivity for CD200, upregulation of CD43 and a weak expression of CD81 are helpful and support the diagnosis of CLL versus mimickers (especially mantle cell lymphoma). Currently, the European Research Initiative for CLL (ERIC) recommends an enlarged panel of monoclonal antibodies (including CD43, CD79b, CD81, CD200, CD10, and ROR1) for the initial characterization, differential diagnosis and follow up of minimal residual disease [21,22].

**Figure 1:**
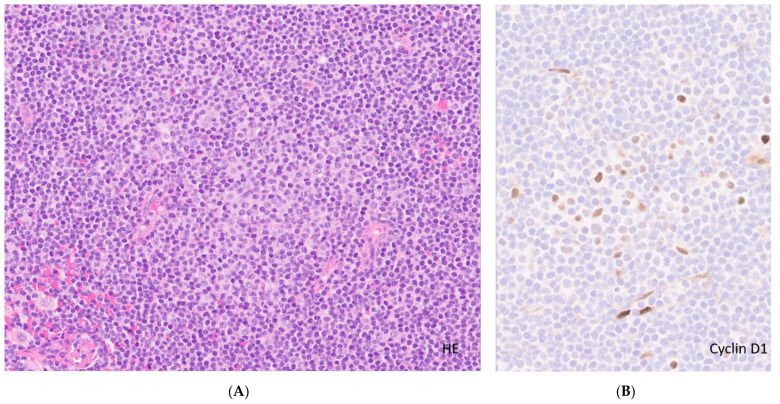
Lymph involved by CLL. (**A**). Lymph node involved in CLL. Effacement of lymph node architecture by neoplastic cells (H&E, original magnification ×10) highlighting a proliferation center (pale area with prolymphocytes/paraimmunoblasts) in the center; (**B**). High-power view of the proliferation center with a few large cells positive for Cyclin D1, (original magnification ×20) contrary to MCL (uniform strong nuclear expression in most cases).

In lymph nodes the disease replaces the normal architecture by a vaguely nodular pattern commonly created by the presence of proliferation centers enriched in prolymphocytes and paraimmunoblasts (paler areas) surrounded by the characteristic small, mature lymphocytes with a clumpy chromatin. In some cases, the proliferation centers may be predominant, forecasting a more aggressive course [23]. The presence of cells expressing Cyclin D1 in the proliferation centers may lead to the confusion with MCL (Figure 1). However, in these cases, the Ig/*CCND1* translocation is absent and the expression of Cyclin D1 is typically weak and limited to a fraction of the neoplastic cells (usually less than 30%). Some cases may exhibit plasmacytoid differentiation, representing an additional diagnostic challenge to distinguish from other similarly differentiated and small B-cell lymphomas. Bone marrow infiltration may be diffuse, nodular or interstitial but paratrabecular deposits, classical for follicular lymphoma, are very rare; proliferation centers are also not common. The splenic involvement is cytologically similar to the description above, and typically expands the white pulp, correlating with grossly white-tanned small nodules. In advanced cases, the disease extends to the red pulp and may become diffuse upon transformation.

Modern genetic technologies led to an improved understanding of the biology of CLL/SLL and to important progress in prognostic stratification. The frequency and impact of recurrent chromosome gains and losses emerged 20 years ago and include, in decreasing frequency, del13q14, trisomy 12, del11q23.3-23.1 and del17p13 [24]. The poor outcomes of patients harboring del17p13 and del11q23.3-23.1, when treated with chemotherapy and immunochemotherapy (ICT), were recognized early and largely confirmed in multiple studies [24,25,26]. Some genetic alterations correlate not only with the outcomes but also with the specific clinical presentations. Trisomy 12 is more frequently found in SLL than CLL and is associated with specific mutational patterns, phenotypic features and clinical outcomes [27]. Cases with del11q23.3-23.1 often present with bulky lymphadenopathy in younger patients [28]. These subtypes may thus be more prone to a lymphomatous presentation including splenomegaly and should be included in the differential diagnosis of a splenic lymphoma.

The analysis of the mutational status of the immunoglobulin-variable heavy chain (IGVH) in CLL largely superseded flow cytometry (CD38 and ZAP70) prognostication and distinguishes two biologically relevant subtypes with different outcomes. Unmutated cases (with more than 98% homology to the germline sequence) follow a more aggressive course compared to mutated cases [29,30]. Furthermore, DNA methylation patterns now identify three disease subgroups, with diverse clinical courses and responses to treatment, which show good correlations with the IGVH mutational status [31,32]. These three distinct epigenetic groups recapitulate normal antigen-driven, B-cell differentiation stages, namely: naive B-cell-like CLL (n-CLL), memory B-cell-like CLL (m-CLL) and intermediate B-cell-like CLL (i-CLL), corresponding to an unmutated, mutated and a non-conforming third group based on VH usage, somatic hypermutation and imprinting analysis, respectively [28]. Further clinical heterogeneity may be explained by specific stereotypes. In fact, approximately 30% of CLL cases have a very limited diversity in the CDR3 regions of the immunoglobulin light- and heavy-chain-variable regions, suggesting a common antigen driver, and can be assigned to disease groups with specific clinical behaviors. These in turn are related to genetic alterations and IGVH mutational status [28,33]. More recently, extensive studies characterizing the genomic landscape of CLL/SLL identified multiple additional mutations [34,35,36]. Although only a few are relatively frequent, their putative prognostic value was extensively investigated [28,37]. Among the most relevant are TP53 mutations, commonly present at relapse and with a strong prognostic and therapeutic impact [25].

CLL/SLL cases presenting with splenomegaly do not have specific flow cytometric or molecular patterns regarding the markers described above.

### 3.4. Differential Diagnosis

The detection of cell surface markers relies on the sensitivity of the techniques employed. The progress in immunohistochemistry and flow cytometry, including improvements in hardware, software and reagents resulted in enhanced sensitivity. Therefore, the frequency of CD5 expression may vary within the same tumor type and between anatomical locations. The importance of an integrated diagnosis based on clinical, morphologic, immunophenotypic and genetic characteristics cannot be overemphasized.

Aside from mantle cell lymphoma (MCL), discussed in detail in the following section, prolymphocytic leukemia (PLL), lymphoplasmacytic lymphoma (LPL), splenic marginal zone lymphoma (SMZL) and hairy cell leukemia (HCL) may be positive for CD5. In addition, aggressive lymphomas may be dominated by splenomegaly and are occasionally positive for CD5. These entities will be briefly discussed at the end of the paper.

### 3.5. Prognosis and Treatment

Clinical staging according to the Ray and Binet systems, once the mainstay of patient stratification, is now less relevant in anticipating the clinical course of CLL/SLL. Age and comorbidities continue to strongly influence patient outcomes. Important predictors of unfavorable outcomes include TP53 alterations (deletions and/or mutations), an unmutated IGVH and elevated beta 2 microglobulin. Multiple prognostic scores were developed. The CLL International Prognostic Index [38] (CLL-IPI), which includes the three previously mentioned variables, was extensively validated [38] and discriminates patient groups with significantly different outcomes in the immunochemotherapy (ICT) era. However, with the generalized use of targeted agents, the importance of these variables needs reassessment. A recent publication analyzed the relevant prognostic factors in patients receiving the BTK inhibitor, Ibrutinib [39]. Further work is needed to identify biomarkers for patients receiving BCL2 inhibitors with or without anti-CD20 monoclonal antibodies and other new agents and combinations under development.

Minimal residual disease (MRD), as detected by either flow cytometry, conventional PCR or Next Generation Sequencing/NGS, negatively impacts the prognosis of patients treated with ICT [40,41] and anti BCL2-containing combinations [42]. To adequately interpret the information provided by tests used to detect MRD, a thorough standardization effort is in progress by cooperative groups such as ERIC [21]. Probably the most useful prognostic scores at the present time are those based on dynamic models considering both the individual patient and disease characteristics, as well as the quality and depth of response to treatment, such as the Continuous Individualized Risk Index proposed by Kurtz et al. [43].

The criteria to initiate treatment in this indolent and still incurable malignancy are well-defined by the iwCLL and relate to the presence of symptoms and/or progressive disease. It cannot be overemphasized that asymptomatic, clinically stable patients should be kept under observation until therapy is needed. The determination of the mutational status of IGVH and TP53 is mandatory for a well-informed choice. Other clinical and laboratorial factors, including beta 2-microglobulin, should be evaluated before treatment decisions [15].

Since 2014, the rapid introduction to the therapeutic armamentarium of agents targeting the BCR signaling machinery (BTK and PI3K inhibitors) and BCL2 inhibitors (Venetoclax) changed the treatment landscape of CLL, initially for relapsed/refractory disease and currently for first line therapy. Immunochemotherapy, once the mainstay of treatment, now has limited indications but may still be worthy of consideration for patients younger than 65 with an IGVH mutated status [25]. The choice of treatment depends on patient age, comorbidities, preferences and goals of treatment, as well as the genetics of the disease. Patients with disrupted TP53 are resistant to ICT and must receive novel agents. IGVH unmutated cases usually lack durable responses to ICT. A survival advantage was shown for young IGVH unmutated patients receiving first-line BTK inhibitors [44]. The choice between different novel agents and therapeutic regimens also depends on their toxicity profiles, which help tailor personalized treatment. Immune therapies, including chimeric antigen receptor T cells (CART), are also being studied for CLL with promising, although still preliminary, results [45].

Despite substantial achievements, important challenges persist for the treatment of CLL, which are the focus of future research. Whether ICT still has a role in selected, low genetic risk fit patients remains unclear. Both BTK and BCL2 inhibitors, with or without anti-CD20 monoclonal antibodies, are approved in many countries. However, due to the absence of comparative data, firm therapeutic guidelines at each phase of the disease are lacking. Moreover, the combined double (BTK and BCL2 inhibitors) and triple (with the addition of anti-CD20 antibodies) therapy was shown to be extremely potent when indirectly compared to single agents, inducing complete responses and MRD negativity that persisted even after therapy cessation [46,47,48,49,50]. Nevertheless, there are no data comparing these combinations to the sequential use of single agents in terms of efficacy, toxicity, costs and the induction of resistance.

## 4. Mantle Cell Lymphoma (MCL)

### 4.1. Definition

MCL is a mature B-cell non-Hodgkin lymphoma (NHL) representing 3% to 10% of the systemic B-cell NHL [2]. The disease is defined in the current World Health Organization (WHO) classification as a “B-cell neoplasm generally composed of monomorphic small-to-medium–sized lymphoid cells with irregular nuclear contours and a CCND1 translocation” [2].

Although traditionally considered a very aggressive lymphoma, two indolent variants are now recognized [2]: in situ mantle cell neoplasia and leukemic, non-nodal mantle cell lymphoma (SOX11 negative), which shows a high prevalence of splenic involvement.

### 4.2. Clinical Features

MCL more commonly affects the elderly and is predominant in males (male to female ratio of 2:1). Most patients present at advanced stages, with lymphadenopathy, Waldeyer ring and extra-nodal involvement, namely hepatosplenomegaly, bone marrow and gastrointestinal tract infiltration (frequently in the form of multiple lymphomatous polyposis). B symptoms may occur [51]. Peripheral blood involvement, with a variable lymphocytosis, is common, not only in the leukemic non-nodal form but also in classical MCL. The identification of PB involvement may require sensitive techniques, e.g., flow cytometry [52], in non-leukemic cases.

### 4.3. Microscopy, Immunophenotype and Genetic Profile

Classical mantle cell lymphoma is a monomorphic proliferation of small- to medium-sized cells resembling centrocytes, with dispersed chromatin and inconspicuous nucleoli. Four variants are recognized: two of them, pleomorphic and blastoid, are associated with an aggressive clinical course. The other two, small cell and marginal zone-like, may have a better prognosis. Nevertheless, only a moderate diagnostic agreement in cytological assessment was shown between labs [53]. Hyalinized, small caliber vessels are common, as well as scattered epithelioid histiocytes [54]. True plasmacytic differentiation is very rare and mainly occurs in the SOX11-negative subtype [55].

The growth pattern is usually vaguely nodular but mantle zone, diffuse, and follicular patterns may be present, sometimes in the same sample [55].

The two more aggressive variants of MCL are characterized by increased mitotic index (usually above 10 per 15 high-power fields). The blastic form shows larger uniform nuclei with fine chromatin, and the pleomorphic form displays more irregular, enlarged nuclei.

**Figure 2:**
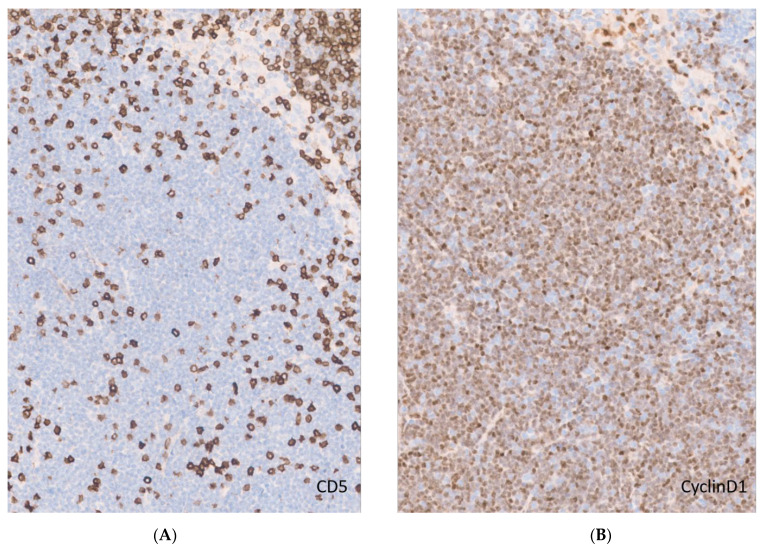
CD5-negative MCL. (**A**). CD5 expression in a lymph node with MCL. Notice the scattered CD5-positive T cells and lack of expression by neoplastic mantle cells (original magnification ×20); (**B**). Cyclin D1 immunohistochemistry demonstrating characteristic diffuse nuclear expression in MCL (original magnification ×20).

**Figure 3:**
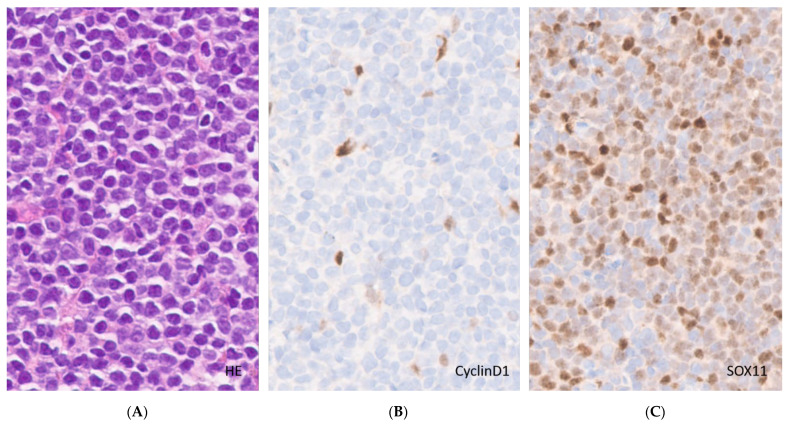
Cyclin D1-negative MCL. (**A**). White pulp expansion by monomorphous neoplastic cells (H&E, original magnification ×40); (**B**). Cyclin D1 immunohistochemistry staining endothelial cells and histiocytes only (original magnification ×40); (**C**). SOX11 immunohistochemistry, nuclear staining of the tumor cells (original magnification ×40).

The immunophenotype of MCL includes the expression of pan B-cell antigens such as CD19, CD20, CD79a and PAX5, together with surface IgM/IgD. Similar to CLL, CD5 is expressed but, in contrast, CD23 is usually negative. The absence of CD5 was reported in 5% to 17% of cases of MCL [56,57] (Figure 2). The aberrant expression of BCL-6 can be seen in about 10% of cases. CD10 coexpression is rarely seen and MUM1/IRF4 positivity is infrequent [58]. Nonetheless, the presence of CD10 is more common in blastoid variants [59]. The majority of MCL cases (>95%) are positive for Cyclin D1 by IHC but negative cases are described [60]. Most of these harbor cyclin D2 or cyclin D3 rearrangements. In this scenario, SOX11 emerges as a useful diagnostic marker of the disease, since it is considered more sensitive than Cyclin D1 and is positive in 93% to 95% of cases (except in leukemic, non-nodal forms). Therefore, the staining for SOX11 in CCND1-negative CD5 expressing lymphomas is recommended to clarify the diagnosis (Figure 3). However, SOX11 expression is not unique to MCL as it can also be seen in lymphoblastic lymphomas, Burkitt lymphoma, hairy cell leukemia, and T-cell prolymphocytic leukaemia [61,62], highlighting the importance of using complete immunohistochemistry panels in routine hematopathology. In addition, the integration of clinical features is important in the differential diagnosis, since these entities are generally CD5-negative and usually lack prominent or isolated splenomegaly, except for hairy cell leukemia.

Immunoglobulin (IG) genes are clonally rearranged, and variable (V) regions are somatically hypermutated in 15–40% of cases. A biased usage of the IGVH genes supports the possibility of an antigenic drive [63,64,65].

MCL has a distinctive cytogenetic translocation, the t(11;14)(q13;q32) rearrangement, which juxtaposes the *CCND1* gene on chromosome 11 with the immunoglobulin heavy-chain gene (*IGH*) on chromosome 14, resulting in the characteristic overexpression of the protein [66]. Rarely *CCND1* translocates with the IG-light-chain genes [67]. Other cyclins are rarely involved in an IG translocation, and these need to be investigated by FISH [67,68].

TP53 inactivation and the proliferative rate (based on Ki67 nuclear expression) are independent prognostic factors and may be detected by IHC. Therefore, Ki67 and TP53 stains should be performed routinely and the Ki67 score is incorporated in the commonly used biological Mantle cell Lymphoma International Prognostic Index (MIPIb) [69].

Other genetic and epigenetic alterations are emerging, which impact our understanding of the disease [70].

Together with an aggressive morphology (blastoid or pleomorphic variants), TP53 alterations are associated with higher proliferation rates (>30%) and worse overall survival in MCL [71,72]. In a study of 59 patients, the median survival was 50 months for patients with conventional MCL and 18 months for those with blastic features.

### 4.4. Prognosis and Treatment

The prognosis of MCL is the poorest among small-cell, B-cell lymphomas, but some subgroups of patients can survive for years with little or no treatment. For example, SOX11-negative, leukemic, non-nodal patients have a more favorable prognosis in the absence of TP53 alterations [73,74,75].

Adequate patient stratification, the usage of aggressive ICT regimens in young patients and rituximab maintenance together with new agents at relapse contributed to improved survival rates. Nevertheless, the long-term prognosis is still poor [76].

Treatment of MCL should take into consideration the biology and behavior of the disease (i.e., the indolent versus aggressive form), the age and fitness of the patient and the phase of the disease (first presentation versus relapse). Although the majority of MCL patients pursue an aggressive clinical course requiring immediate treatment, several investigators identified indolent forms of disease representing 10–15% of cases [34]. As mentioned above, these include not only the leukemic/non-nodal, SOX11-negative form but also rare, asymptomatic, nodal/extra-nodal forms with a low tumor burden, classical morphology on histopathology, low proliferation rate and an intact TP53 [51,76]. It was shown that these subsets may remain stable for years and that early treatment does not impact overall survival [77,78]. Therefore, it is currently recommended to keep these patients under surveillance, starting treatment only when symptoms or clear progression demands it.

The mainstay of first-line treatment for MCL patients still relies on ICT and includes an induction, a possible consolidation and a maintenance regimen. Young, fit patients should receive induction regimens including Rituximab and Cytosine Arabinoside [73,79,80] and be consolidated with high-dose chemotherapy and autologous stem cell transplants, which has recently been shown to prolong OS [79,80,81]. The addition of an extended course of Rituximab maintenance further prolongs OS and should be prescribed [80]. Elderly or unfit patients commonly receive RCHOP, R Bendamustine or VR CAP [51,82] without consolidative transplant. In a randomized trial, Rituximab maintenance after RCHOP was shown to significantly prolong OS [83] and retrospective analyses suggest that the same may be true after R Bendamustine [84].

Despite considerable progress in the treatment of MCL with consequent improvements in survival, the disease remains incurable, and relapses are inevitable. Targeted therapies approved for relapsed refractory MCL include the proteasome inhibitor Bortezomib, the immune modulator Lenalidomide and, in Europe, the mTOR inhibitor Temsirolimus [85,86]. However, very active BTK inhibitors (BTKi), of which Ibrutinib [78,85,87] and Acalabrutinib [88] are approved, currently dominate the therapeutic strategies. Numerous clinical trials are testing the combination of new agents with chemotherapy at the first line, the addition of lenalidomide or bortezomib to maintenance regimens and the use of new agents in comparison to chemotherapy [72,76]. The future inclusion of BTKi in first-line regimens will change the treatment of relapsed disease. The efficacy of next generation, non-covalent BTKi, BCL2 inhibitors and new anti-CD20 monoclonal antibodies, alone or in combination, is under investigation and may prove useful in patients with high-risk genetics [72]. Perhaps the most attractive option nowadays for multiple-relapsed, BTKi-resistant patients is the recently approved anti-CD19 CART which led to very high ORR and CR (92% and 67%, respectively) in a phase 2 trial [89]. Other CART products are under investigation (NCT02631044). The prolonged disease control seen in these very poor prognosis patients holds significant promise, although a mature follow up is needed.

### 4.5. Differential Diagnosis

A recent review [90] expanded the earlier observation [91] that MCL may mimic extra-nodal, marginal-zone, B-cell lymphomas (EMZL). These cases share the morphologic and phenotypic features of the nodal MCL and the indolent course of the indolent variant. “EMCL” may be a subset with a preferential involvement of Waldeyer’s ring and gastrointestinal tract, showing clinicopathologic overlap with EMZL.

The expression of CD5 can be seen in up to 10% to 15% of cases of DLBCL [92], and also in intravascular B-cell lymphomas. However, intravascular lymphomas have a very peculiar intravascular compartmentalization, absent in MCL. Furthermore, rare cases of DLBCL can be associated with Cyclin D1 expression [93]. In this respect, none of those cases show the *CCND1* rearrangement seen in MCL, a feature that, together with a larger cell size, usually allows to distinguish them from MCL.

The blastoid or pleomorphic variants of MCL may also be confused with B-lymphoblastic leukemia/lymphoma (B-ALL). In contrast with B-ALL, only rare cases of MCL are TdT positive [94] and the immaturity markers, CD34 and CD99, are absent in MCL, allowing a diagnostic clarification.

The leukemic, non-nodal form of MCL commonly presents with splenomegaly and lymphocytosis without lymphadenopathy or with only a few small, enlarged lymph nodes [95]. This presentation can be misdiagnosed as CLL. However, peripheral blood phenotyping (see above) and FISH for *CCND1* t(11;14)(q13;q32) should allow the correct diagnosis. Other entities presenting with lymphocytosis and splenomegaly that may occasionally express CD5 are discussed in the following sections and covered in more detail in the companion articles in this issue.

## 5. Lymphomas Rarely Expressing CD5

Other lymphomas in the spleen rarely expressing CD5 include B-cell prolymphocytic leukemia (PLL), now a controversial entity; lymphoplasmacytic lymphoma (LPL); splenic marginal zone lymphoma (SMZL) and hairy cell leukemia (HCL). The main distinctive features are depicted in Table 1. Occasionally, the clinical picture of CD5-positive, aggressive lymphomas may be dominated by splenomegaly. However, characteristic morphologic features, including large cells, allow definite categorization.

### 5.1. Prolymphocytic Leukemia

PLL is a rare (1% to 3% of all leukemias) and aggressive leukemia that may occasionally pose differential diagnostic problems with CLL, when derived from a B-cell lineage. It usually manifests quite acutely with systemic symptoms, massive splenomegaly and high-count lymphocytosis; cytopenias and lymphadenopathy may also occur [1,96]. By flow cytometry B-PLL expresses bright, pan-B-cell markers including CD19, CD20 (usually brighter than in CLL), CD22, CD79a, CD79b and restricted surface immunoglobulin light chains. CD5, CD200 and CD23 are variably present. In common with classical HCL (but not variant HCL), CD11c is frequently expressed. However, CD25, CD123 and CD103 are absent, excluding classical HCL. The genetic background of the disease is not completely defined and so far, the alterations are unspecific. Frequent defects in TP53 (75%) and MYC (50%) occur [97,98], justifying chemoresistance and, currently, the therapeutic use of BCR or BCL2-inhibitors when TP53 is disrupted [99,100]. The impact of these inhibitors on the poor clinical outcome of B-PLL remains to be fully clarified.

### 5.2. Lymphoplasmacytic Lymphoma

LPL may present with splenomegaly in addition to hepatomegaly and lymphadenopathy. The hallmark of this disease is, however, a predominantly diffuse BM infiltration by lymphocytes, some with plasmacytoid differentiation, and plasma cells, all belonging to the neoplastic clone. Therefore, BM is the preferred diagnostic material in LPL [101]. The morphology may, however, be insufficient to distinguish between various BM-infiltrating, low-grade, B-cell lymphomas, and phenotypic/genetic characterization is an important part of the diagnostic workup [102]. LPL cells express the B-cell makers CD19, CD20, CD79a, CD22 (dim), CD25 and CD27 and are classically IgM positive, but are usually negative for CD5, CD10, CD23 and CD103. The expression of CD5 is described in up to 20% of cases [103], which may present as a monoclonal B lymphocytosis raising the possibility of CLL/SLL or MCL. However, the correct diagnosis is suggested by both the morphology and clinical features, with variable combinations of cytopenias (mostly anemia) and the frequent production of an IgM monoclonal paraprotein defining Waldenstrom’s macroglobulinemia (WM) with associated manifestations of hyperviscosity, peripheral neuropathy, cutaneous alterations, autoimmune phenomena and cryoglobulinemia [104].

The genetic findings also contribute to the differential diagnosis. The translocation of t(11;14) (q13;q32) is absent, generally excluding MCL. The detection of the MYD88^L265P^ mutation, present in more than 90–95% of all WM cases [105,106], is very helpful. Of note this mutation can be found in other low-grade, B-cell lymphomas, including 5–10% of marginal-zone lymphomas and even in CLL/SLL [107], requiring the finding to be interpreted in the setting of clinical and morphological information. Additionally, 30–40% of WM patients have mutations in the CXCR4 gene. In contrast to MYD88, more than 40 types of CXCR4 mutations are described, complicating diagnostic standardization [108].

The outcome and treatment of WM, an indolent but incurable disease, is nowadays influenced by the genomic characteristics [109]. First-line treatment is still ICT-based in many parts of the world, although BTKi are highly efficient [110]. The therapeutic choices should consider tumor bulk, dominant clinical manifestations and patient fitness [111]. Patients requiring urgent control of hyperviscosity, cryoglobulinemia or hemolytic symptoms should initially undergo plasmapheresis. The BTKi Ibrutinib demonstrated efficacy in MYD88L^MUT^ and CXCR4^WT^ patients [108], and the addition of Rituximab may overcome the Ibrutinib resistance associated with CXCR4 mutations [112]. The emerging biologic understanding of WM provides grounds for developing several new therapeutic agents and combinations [105].

### 5.3. Splenic Marginal Zone Lymphoma

SMZL is another uncommon subtype of indolent B cell non-Hodgkin lymphoma/NHL (<2% NHL), typically presenting with splenomegaly and BM infiltration [113]. Although splenic hilar lymph nodes are commonly involved, the disease usually spares more distant nodes and organs. Cytopenias attributable to hypersplenism and BM infiltration are common. A fraction of patients has autoimmune manifestations and up to one third may produce a serum monoclonal protein [1,114,115]. Approximately 10 to 16% of patients may have an associated Hepatitis C infection, which may constitute an antigenic trigger for lymphomagenesis [116]. Occasionally, isolated lymphocytosis can be present and may remain stable for prolonged periods or progress into a full-blown SMZL. These cases seem to have a favorable outcome and are now called clonal B-cell lymphocytosis with marginal-zone phenotypes [1,115,117].

The diagnosis is suggested by the presence of small lymphocytes with short cytoplasmic projections. The phenotypic characterization of BM and/or PB is again helpful in the differential diagnoses. Neoplastic cells express the common B-cell markers CD20, FMC7, CD19 and CD79a, and, frequently, surface IgM and IgD. In contrast, CD5, CD10 and CD23 are usually negative. Importantly, 20–25% SMZL cases may express CD5. The absence of Cyclin D1 and CD23 helps to differentiate SMZL from MCL and CLL, respectively [9]. The bright expression of CD20 and the surface light chains and the positivity for FMC7 further distinguish SMZL from typical CLL. The CD5-positive cases have a higher lymphocytosis [113,118] and seem to correlate with distinct morphological (predominance of prolymphocytic cells), cytogenetic (translocations involving the CDK6 cyclin) and molecular (*TP53* mutations) alterations [119].The classical translocations seen in MALT lymphomas are absent, but a fraction of cases show deletions of chromosome 7q (the most common aberration) and alterations in chromosomes 3q, 1, 8 and 14; however, these findings are not specific [120]. Deep genomic analyses identified *NOTCH2* and *KLF2* mutations as the most common alterations in SMZL, but again those are not entirely specific and, more importantly, are not yet searched for in routine clinical practice despite their potential clinical and prognostic implications [121].

When available, spleen histology allows the diagnosis by showing a predominant involvement of the white pulp, with small, round lymphocytes invading the germinal centers and mantle zone and merging with small- to medium-sized marginal zone cells with irregular nuclei and more abundant pale cytoplasms (monocytoid differentiation), as well as variable numbers of larger centroblasts and immunoblasts [1]. Staining for IgD and Ki67 may be helpful for identifying white pulp expansion, typically highlighting a targetoid pattern.

The treatment of SMZL once included splenectomy as a first option in most cases. In recent years, Rituximab, alone or with chemotherapy, also led to favorable outcomes [115], sparing this elderly population the possible complications of surgery. At the same time, this shift in treatment reduced the availability of the splenic tissue for analysis and the diagnosis mostly relies on BM and PB examination, in the appropriate clinical setting. Treatment of an underlying Hepatitis C, when present, should be attempted since it can lead to lymphoma remission [122].

Although incurable, SMZL patients have globally favorable outcomes with a median overall survival exceeding 10 years. Outcomes may be impacted by the mentioned genetic features and a small (5–10%) but noticeable risk of transformation to DLBCL [123].

### 5.4. Hairy Cell Leukemia

HCL is a very rare (<1% of lymphoid neoplasms) form of leukemia with an indolent course, affecting predominantly middle-aged Caucasian males. Patients present with splenomegaly and pancytopenia, without lymphadenopathy in most cases [124]. Fatigue caused by anemia and frequent infections are common symptoms. Bulky splenomegaly, when present, may be symptomatic and suggest a chronic myeloproliferative neoplasm. PB counts show a characteristic monocytopenia and the smear reveals small- to medium-size tumor lymphocytes with oval nuclei; an abundant, pale cytoplasm and circumferential villous projections, which give the disease its name [2]. Tumor cells are present in variable quantities in BM aspirates, which tend to be paucicellular (“dry tap”), and are typically abundant in BM biopsy, showing a characteristic “fried egg” appearance and growing in an interstitial pattern. Fine reticulin fibrosis surrounding each cell is characteristic and contributes to the unyielding nature of BM aspirates. Phenotypic confirmation is obtained by flow cytometry and immunohistochemistry, showing a bright expression of CD19, CD20, CD22 and CD200, and at least three of the four classical markers CD25, CD11c, CD103 and CD123 (variable). The detection of Annexin A1, TRAP, BRAF and CD72/DBA.44 by IHC may contribute to the diagnosis. The expression of CD5 is exceedingly rare but was described in up to 2% of cases [125,126,127]. Together with the positivity for Cyclin D1 in half of the patients and the possible presence of lymphadenopathy, CD5-positive cases can be misdiagnosed as MCL. The absence of t(11,14) q(13;q32) by FISH and the presence of BRAF V600E mutations help to clarify the diagnosis.

BRAF V600F mutations, as suggested by immunohistochemistry and confirmed by PCR, support the diagnosis of HCL in cases where flow cytometry is unavailable [128]. MRD at the end of treatment was usually detected by flow cytometry or BRAF PCR; however, IHC is now available [129]. Since the late 1980s, chemotherapy with purine analogues for symptomatic patients and those with declining CBCs is the mainstay of HCL treatment. Overall and complete response rates exceed 95% and 75%, respectively [130,131]. Although responses are durable, half of the patients relapse at 5 years. Higher CR rates with undetectable MRD may be achieved by combining Rituximab [132,133] and by using new agents, including immunoconjugates targeting CD22 [134], BRAF and MEK inhibitors [135,136] and, more recently, BCR inhibitors and Venetoclax [137]. The combination of the BRAF inhibitor Vemurafenib with Rituximab was shown to increase the negative, durable CR responses of MRD in relapsed/refractory patients [138]. Recently identified clinical and molecular prognostic factors may help to better stratify these multiple new therapeutic options in the future [137].

### 5.5. Aggressive B Cell Lymphomas

Aggressive lymphomas presenting in the spleen include DLBCL, the T-cell/histiocyte-rich, B-cell lymphoma (THRBCL) variant and intravascular, large, B-cell lymphomas.

#### 5.5.1. Diffuse Large B Cell Lymphoma

DLBCL can rarely (<1% of all lymphomas) present as an isolated splenic mass [139]. These cases are commonly diagnosed on splenectomy or in spleen biopsy specimens. Approximately 5 to 10% of DLBCL expressed CD5. Single or multiple splenic tumors may be present, and the histological appearance is similar to nodal or extranodal DLBCL. White pulp involvement is the norm but occasionally the red pulp may be predominantly involved [1,2,140]. Splenic hilar lymph nodes are commonly affected and may be sampled for histological analysis. In contrast, dissemination to other organs is rare and the prognosis is rather favorable, especially given that ICT supplements surgery [140]. A short course of RCHOP-like chemotherapy was recently reported in localized DLBCL with expected excellent outcomes [141,142].

#### 5.5.2. T Cell/Histiocyte-Rich Large B Cell Lymphoma

THRBCL comprises less than 10% of all DLBCL. When the spleen is involved, the BM, liver and lymph nodes are usually infiltrated as well. The disease is characterized by rare (less than 10%) and large neoplastic B cells without sheet formation in a background of abundant T cells and histiocytes [143]. The expression of CD5 is very unusual [144].

Occasionally, patients may present with an isolated splenomegaly or have additional small lymph nodes not readily accessible to biopsy. In these rare cases, a BM biopsy may provide the diagnosis, revealing the infiltration by neoplastic cells. The interpretation of BM biopsy can be hindered by the characteristic, extensive inflammatory T cell infiltrate [145], requiring a diagnostic splenectomy.

#### 5.5.3. Intravascular Large B Cell Lymphoma

Intravascular large B-cell lymphoma, another rare subtype of aggressive B-cell lymphoma, may also uncommonly express CD5 [2]. The hallmark of this entity is the intravascular growth of large CD20-positive B cells inside small- to medium-sized vessels in the affected organs [146]. The classical form affects mainly BM, the central nervous system and skin, while Asian patients present more often with hepatosplenomegaly and hemophagocytosis [147]. Trephine BM biopsy is usually diagnostic, even when splenomegaly happens to be the manifestation driving the clinical investigation. The outcomes of intravascular large B-cell lymphoma are poor. Although treatment is not standardized, an anthracycline-rituximab-based regimen is usually recommended and central nervous system prophylaxis should be added when possible [148].

## 6. Clinical Vignette–Part 2

PB flow cytometry in our patient showed the presence of a CD19/CD79b positive mature B-cell population with a monoclonal surface lambda-restricted pattern and bright CD20 expression. The CD10, CD23 and CD200 surface markers were absent. CD5 was strongly and homogeneously expressed. Although flow cytometry findings were supportive of MCL over CLL, confirmatory tests were necessary. FISH analysis performed in PB showed the presence of t(11;14) (q13;q32) translocation. The clinical picture suggested an indolent, non-nodal form of MCL. BM trephine biopsy revealed an interstitial infiltration by small CD20-positive/kappa negative B lymphocytes. SOX11 was negative and the Ki67 proliferation rate was only 5%. A diagnosis of mantle cell lymphoma with classical cytology was made.

Staging CT scans of the cervical, thoracic, abdominal and pelvic regions revealed small, <2 cm retroperitoneal lymph nodes and a homogeneous, non-nodular splenomegaly of 17 cm. Further cytogenetic and molecular characterization in the BM aspirate showed a hypermutated IGVH and the absence of del17p and TP53 mutations. The first of these findings further supported the diagnosis of leukemic non-nodal MCL. An intact TP53, together with a low Ki67 and classical morphology also supports a low-risk disease [149]. Following discussions at the multidisciplinary team meeting, PET-CT and gastroscopy/colonoscopy examinations were deemed unnecessary, since the patient was asymptomatic. Given the expected indolent behavior of this form of MCL, a watch and wait strategy was adopted. After 22 months, the patient remains asymptomatic with stable CBC counts, as well as lymphadenopathy and splenomegaly on CT scans.

## 7. Conclusions

The involvement of the spleen by CD5-expressing, B-cell neoplasms usually corresponds to CLL or MCL. The differential diagnosis includes indolent leukemias and lymphomas that less often express CD5. Large B-cell lymphomas very rarely manifest as an isolated splenomegaly.

Most splenic lymphomas also involve BM and PB, even if not in a prominent form, allowing a specific diagnosis without resourcing to splenectomy. However, the surgical removal of the spleen is indicated when an aggressive lymphoma cannot be excluded, due to significant therapeutic implications.

The final diagnosis relies on the integrated analysis of histomorphology complemented by phenotypic and genetic findings in the appropriate clinical setting:

- CLL and MCL, as well as HCL, have a characteristic constellation of phenotypic features in most cases.
- The presence of more than 55% of prolymphocytes in PB smears suggests prolymphocytic leukemia.
- A morphological continuum between lymphocytes, lymphoplasmocytes and plasma cells in BM is found in LPL/WM and other lymphomas with plasmacytic differentiation.
- SMZL remains a diagnosis of exclusion sharing clinical and morphological features with LPL and having a non-characteristic immunophenotype.
- The genetic tests commonly accessible to the practicing clinician contribute to the differential diagnosis in MCL, HCL and LPL, where the t(11;14) (q13;q32), BRAF V600E and MYD88^L265P^ mutations are the rules, respectively. Deeper genetic studies should be undertaken for diagnostically difficult cases, and when findings contribute to critical prognostic stratification and therapeutic choices, such as in CLL.

## Figures and Tables

**Table 1:** Differential diagnosis of splenic CD5-positive indolent lymphomas.

	CLL	cMCL	nn MCL	PLL	HCL	LPL	SMZL
Clinical features	Lymphocytosis, lymphadenopathy, cytopenias, occasional autoimmune phenomena	Adenopathy, splenomegaly, frequent BM and GI infiltration, possible lymphocytosis	Lymphocytosis, splenomegaly without lymphadenopathy	Massive splenomegaly, rapidly raising lymphocytosis, systemic symptoms	Splenomegaly (occasionally bulky) without adenopathy; cytopenias, fatigue, opportunistic infections	Hepatosplenomegaly, lymphadenopathy, cytopenias, hyperviscosity, neuropathy, cryoglobulinemia, hemolytic anemia	Splenomegaly, cytopenias, variable lymphocytosis rare adenopathy, autoimmune phenomena
PB smear	Small round lymphocytes, scant cytoplasm, clumpy chromatin, smudged cells	Small lymphocytes with irregular nuclei (classical forms); variants with pleomorphic and blastoid cells (10%)	Small lymphocytes with irregular nuclei	>55% prolymphocytes	Small/medium lymphocytes with abundant pale cytoplasm and regular villous projections	(Occasionally) small lymphocytes, lymphoplasmocytes and plasma cells	Small lymphocytes with short, polar cytoplasmic projections (villous lymphocytes)
Phenotype	CD5-positive with CD20 ^dim^ light chain ^dim^, CD79b ^dim/negative^, CD23, CD200 and CD81 positove CD10 negative FMC7 negative	CD5-positive with CD19 + CD20 ^bright^, CD22 ^bright^, light chains ^bright^ CD79b ^bright^ CD23 CD200 and CD10 negative all except CLL FMC7 positive	Same as cMCL	Bright CD19, CD20, CD22, CD79a/CD79b and surface light chains. Variable CD5, CD200, CD23	Bright CD19, CD20, CD22 and CD200. Annexin A1 and CD72 At least 3 of the following: CD25, CD11c, CD103 and CD123	CD19-, CD20-, CD79a-, CD22^dim^-, CD25- + CD27-positive IgM-positive. Rare CD5-positive. CD10-, CD23-, CD103-negative	CD20-, CD19-, CD79a- and surface IgM- and IgD-positive. CD10- and CD23-negative CD43 is usually positive in all low-grade B-cell lymphomas except Follicular
BM histology	Diffuse or nodular infiltration by small lymphocytes; proliferation centers; paraimmunoblasts	Small lymphocytes with irregular nuclei in classical forms, Pleomorphic and blastoid cells forms in 10% cases	Small lymphocytes with irregular nuclei, dispersed chromatin, occasionally prominent nucleoli.	Interstitial or nodular infiltration by prolymphocytes	Interstitial infiltration by widely spaced tumor cells, “fried egg” appearance, positive Increased reticulin.	Diffuse, nodular and/or interstitial infiltration by small lymphocytes, lymphoplasmacytes and plasma cells. Mast cells frequently increased	Sinusoidal or nodular small lymphocytic infiltrate
Distinctive genetic findings	FISH panel * IGVH mutational status TP53 mutations	t(11;14)(q13;q32). Additional alterations (DNA repairing including TP53 mutations, cell cycle regulators, apoptosis). Genomic complexity. IGVH-unmutated	t(11;14)(q13;q32) Few additional genetic alterations. IGVH-mutated. Possible acquisition of TP53 and other poor prognosis mutations	TP53 mutations (75%). MYC abnormalities (50%)	BRAF V600F mutations (>90–95%)	MYD88^L265P^ mutation (>90%). CRXC4 mutations (30–40%)	Del7q (25–40%), NOTCH2 (20–40%) and KLF2 (30–40%) mutations. Possible TP53 mutations
Spleen histology	Mostly white pulp, red pulp also involved. Proliferation centers less common than in LN Plasmacytoid differentiation possible	Expanded white pulp, infiltration of the red pulp possible. Nodules may be large and confluent. Residual germinal centers in 50% of cases. Possible marginal zone-like area at the periphery of the nodules.	Same as cMCL	White pulp nodules, red pulp also involved	Red pulp involvement with tumor cells filling cords. Hairy cells surrounding pooled erythrocytes (blood lakes/pseudosinusoids)	Nodular red pulp infiltration by lymphoplasmacytic cells. Diffuse growth also possible	Nodules in white pulp replacing germinal centers and effacing mantle zones. Marginal zone differentiation with dispersed centroblastic cells. Red pulp also involved with nodules of large cells, and small cells in the sinuses

* FISH panel in CLL identifies del13q14, del11q23.3-23.1, tris 12 and del17p13; CLL Chronic lymphocytic leukemia; cMCL Classical mantle cell lymphoma; nnMCL Leukemic non nodal mantle cell lymphoma; PLL Prolymphocytic leukemia; HCL Hairy Cell leukemia; LPL Lymphoplasmacytic lymphoma; SMZL Spleen Marginal Zone Lymphoma.
